# Metakaolinite Phosphate Cementitious Matrix: Inorganic Polymer Obtained by Acidic Activation

**DOI:** 10.3390/ma12030442

**Published:** 2019-01-31

**Authors:** Antigoni Katsiki, Tobias Hertel, Tine Tysmans, Yiannis Pontikes, Hubert Rahier

**Affiliations:** 1 Department of Materials and Chemistry (Physical Chemistry and Polymer Science), Vrije Universiteit Brussel, Pleinlaan 2, 1050 Brussels, Belgium; hubert.rahier@vub.be; 2 Department of Materials Engineering, KU Leuven, 3001 Heverlee, Belgium; tobias.hertel@kuleuven.be (T.H.); yiannis.pontikes@kuleuven.be (Y.P.); 3 Department of Mechanics of Materials and Constructions, Vrije Universiteit Brussel, 1050 Brussels, Belgium; tine.tysmans@vub.be

**Keywords:** chemically bonded phosphate cements, inorganic polymers, metakaolin, acidic activation, geopolymers

## Abstract

This work aims to study an aluminosilicate phosphate cementitious matrix. The cementitious matrix was studied on paste samples. The synthesis of metakaolinite phosphate cement (MKPC) was investigated using calorimetric techniques. A systematic study was performed by emphasizing a broad range of Al/P molar ratios, covering the different behavior of the material to the extremes, as well as the optimum composition. X-ray diffraction and scanning electron microscopy revealed that the final structure was mainly an amorphous network, albeit with some non-reacted phases. The compressive strength was studied on mortars using a cement/sand ratio of 1:3. MKPC specimens with Al/P ratios close to 1/1 showed optimal behavior. MKPCs with Al/P ratios above 1/1 were characterized by high porosity and low strength, whereas MKPCs with Al/P < 1 contained an excess of phosphates. The influence of the Al/P molar ratio on compressive strength was also studied, reaching a maximum of 68 MPa for the optimum composition. Based on the results, MKPC may be a promising candidate for construction purposes.

## 1. Introduction

Structural concrete has been extensively used in recent decades as the most economical building material. However, in today’s construction sector, the level of innovation and improvement has increased. Because of corrosion issues due to steel reinforcements, as well as difficulties regarding the design of free-form buildings and complex-shape facades, the search for innovative cementitious materials is in the spotlight [1,2,3].

Thus, textile-reinforced cementitious (TRC) composites have gained interest, aiming for precast structures which can be light-weight but durable and fire-resistant at the same time [4,5]. An additional important factor, especially for engineers and architects, is the ease of fabrication and wide-spread design variation.

One of the most attractive advantages of the production of TRCs with the use of glass fiber reinforcement is the reduction of the amount of material, and thus, depletion of transportation weight. Moreover, the use of glass fibers instead of steel reinforcements makes the addition of the protection layer on top of steel bars superfluous. The reduced use of raw materials, as well as the search for alternative cements based on by-products and/or waste, go hand in hand with other crucial factors such as cost efficiency and sustainability.

This paper is an introductory study on a phosphate cementitious matrix which will be used in combination with textile reinforcement to give rise to lightweight TRC structures. Regarding the matrix materials, during recent decades, attention has been given to inorganic polymers as a means of reducing carbon dioxide emissions originating from Ordinary Portland Cement (OPC), and thus, to lower the environmental impact of construction [6,7]. Chemically-bonded phosphates are claimed to be 21st century materials by their inventor Wagh [8]. However, very little literature exists on these types of materials for construction purposes, indicating that little attention has been paid to them.

Phosphate cements are a two-component system, consisting of a hardening liquid and a solid phase. The result is a ceramic material with a 3-dimensional network structure that is strong, durable and fire-resistant. Due to their non-alkaline environment during, and after setting and hardening, they can be combined with glass fiber reinforcement. Therefore, they can be used in many applications where excellent thermal resistance and lightweight construction are demanded.

Different names are given from the research community to this new group of materials: “Phosphoric acid-based geopolymers,” “Chemically bonded phosphates,” or even “Geopolymers” [9]. For ease of reference and homogeneity throughout the scientific community, Wagh proposed the use of the term “chemically bonded phosphate cements, CBPCs” to address products formed from chemical reactions occurring between a solid and a phosphate hardening liquid, at room or near room temperatures. An inorganic calcium phosphate cement (IPC), commercially named Vubonite® [10], that hardens at room temperature and whose setting time can be controlled, has been developed at Vrije Universiteit of Brussels (VUB). After hardening, the pH is totally neutral; thus, the matrix can be combined with relatively low-cost E-glass fibers [11].

Numerous studies have already been performed explaining the important role of metakaolinite as an additional component in Portland cement, as well as its high contribution in concrete [12,13,14,15,16]. Metakaolinite is obtained through the calcination of kaolinite (Al_2_O_3_·2SiO_2_·2H_2_O) at approximately 750 °C, rendering it X-ray amorphous, and thus, more reactive [17,18]. Many different types of kaolinitic clays exist, giving rise to different types of metakaolinites, with variation on reactivity and mechanical properties depending on calcination conditions and the origin of the clay [19]. It has been widely proven that by using kaolinites from different locations, or different ways of calcination, studies on the ratios and particle sizes which give the optimum properties will be needed each time. On that point, there was the interest to study it as the primary raw source to create a new, cementitious inorganic material.

The alkali activation of aluminosilicates has been extensively investigated in the literature [20,21,22]. However, there are only a few articles on acidic activation. Perera et al. [23] showed that phosphoric acid-bonding of metakaolinite could lead to high-strength materials (146 MPa as a paste), with superior strength to alkali activated ones (72 MPa as a paste). They performed a study on the polymerization conditions of the acidic activated inorganic polymer and obtained a phosphate cementitious paste with remarkable strength. They found that 6-fold coordinated aluminium and silicon in different environments probably coupled to Al and P over O-bridges. They concluded that still more research needs to be done to determine the correct structure.

Recent work carried out by H. Tchakoute et al. was also focused [24] on comparing the acidic activated system to the alkali one, demonstrating once more the superiority of the CBPCs in terms of strength. They studied the mechanical and microstructural properties of both systems and addressed the effect of the formation of berlinite (AlPO_4_) in the acidic activated ones. They reported that the formation of AlPO_4_ at ambient temperatures is responsible for the high strength of the acidic activated cement, and it behaves as a binder, thus reinforcing the structure and enhancing the strength. This finding was also reported by Khabbouchi et al. [25]; however, contrary to that of Tchakoute, berlinite was only present at elevated temperatures (750 °C, for 11 wt% P_2_O_5_ and 250 °C for 17 wt% P_2_O_5_).

L. Liu et al. [26] attempted to prepare a novel porous phosphoric acid-based inorganic material from chemosynthetic Al_2_O_3_·2SiO_2_ powders, phosphoric acid, and Al powder as a pore-forming agent. The final produced materials consisted of an amorphous phase with minor amounts of quartz and aluminum phosphate. By changing the fraction of the Al powder as well as of the water, the pore size and pore volume fraction could be controlled. The porosity varied from 40% to 83%; they showed excellent thermal stability and nearly constant compressive strength (>6.0 MPa) or shrinkage (5.3%) by heating to 1450 °C. More recent research findings by Celerier et al. [27,28] confirmed the thermal stability of metakaolinite phosphate cements up to 1400 °C without melting. Moreover, they reported that better fire resistance is achieved when less reactive metakaolinite is used, confirming once more the crucial role of the aluminosilicate precursor in the final product.

Other studies by Louati et al. [29,30,31] addressed the synthesis of phosphate cements based on calcined illito-kaolinitic clay and phosphoric acid. They studied the kinetics of polymerization, as well as the influence of the particle size of the precursor in the reactivity. They reported that the specific surface of the clay particle size has a strong impact on the rate of the reaction, as well as on the formation of the molecular structure.

H. Douiri et al. [32,33,34] performed a thorough study on the thermal and dielectric properties of acidic activated phosphate cements, proving that they can be used as insulating materials. More specifically, they showed that as the amount of the acidic activator increases, the amorphousness of the structure increases as well, giving rise to better ionic conduction, and thus, to a great change in dielectric properties.

This study focuses mainly on the fabrication of chemically-bonded phosphate cement, the so-called metakaolinite phosphate cement (MKPC), its structure, physical, and mechanical properties. MKPCs are mainly amorphous, non-crystalline networks; however, some phases can crystallize during/after hardening, depending on the composition of the starting materials as well as on the mixing and curing conditions (temperature and time). MKPC is not well documented in the literature (for example, there is a lack of information on series regarding compressive strength) and generally, conflicting data are found on stoichiometry and the final structure.

A recent review on aluminosilicate phosphate cements provides a complete overview of the aluminosilicate CBPC studies which have been reported in the literature so far. The raw material characteristics, curing processes, molar ratios used, as well as mechanical properties are highlighted. Mechanical and microstructural properties of the aluminosilicate phosphate cements largely depend on the raw precursor and its chemical and mineralogical composition. The amorphous fraction of the final product is correlated to the mechanical properties of the material. However, most commonly, the authors do not prove this with data. What stands out is the lack of systematic studies based on mechanical properties, especially regarding the ageing of the samples. Moreover, another source of uncertainty is whether researchers use the addition of sand without mentioning it or whether the presented values are always based on pastes. The lack of information regarding the finally formed products, as well as the reaction mechanism behind those systems, is also striking [35].

Below, a systematic study will be presented by emphasizing on a broad range of Al/P molar ratios, covering the different behavior of the material from the extremes to the optimum. As a first step, the stoichiometric ratio will be approximated from calorimetric measurements. The composition will be optimized via the measurement of compressive strength to yield the highest possible strength performance and minimum porosity for structural applications.

## 2. Materials and Methods

Materials used to prepare the inorganic phosphate cementitious specimens were metakaolinite (coded MK) from IMERYS Minerals Ltd., Paris, France (d_50_ = 2 μm) as aluminosilicate precursor, phosphoric acid (H_3_PO_4_ 85% from VWR Chemicals) as an acidic activator, and distilled water for the solution preparations. Pure quartz sand provided by Sibelco Company, Dessel, Belgium (d_50_ = 260 μm) was added as a filler in the metakaolinite and phosphoric acid to synthesize the cement mortars. The chemical and mineralogical compositions of metakaolinite and H_3_PO_4_ are given in Table 1. They were used without any further modifications.

Metakaolinite, phosphoric acid, and distilled water were mixed with sand and a high-shear mixer for about 5 min at a speed of 3600 rpm, to obtain a homogeneous slurry. The Al/P molar ratios ranged between 0.44 to 2.50. Indicatively, the ratio of water to solid precursors, meaning water (extra water + ‘water’ in H_3_PO_4_)/(metakaolinite + anhydrous phosphates), was 0.29 for Al/P = 0.44, 0.43 for Al/P = 0.94, and 0.59 for Al/P = 2.50. Extra water was used to dilute the commercial concentrated phosphoric acid solution. Regarding the commercial phosphoric acid solution, part of the water is not free because it is attached to H_3_PO_4_, but it is released during the reaction; thus, it should be taken into consideration.

In the case of mortars, the ratio of metakaolinite to quartz sand by mass was 1:3. The aqueous slurry was then poured into cylindrical plastic containers and vibrated to expel the air pores. All the specimens were sealed to prevent the evaporation of water. The cylinders were stored for one day at room temperature and then post-cured at 60 °C for 24 h. For the characterization of the microstructure, the paste was also cast into plastic molds and cured in the same way. The optimum composition was chosen based on both calorimetric and mechanical testing experiments, and in accordance with specific requirements such as rheology and workability. Regarding the room temperature cured specimens, they were sealed and cured at room temperature and 60% relative humidity.

To characterize the developed cementitious material, the pH, the microstructure and mechanical properties were measured. The DSC experiments were performed on a Mettler Toledo DSC822e (Columbus, OH, USA) with mechanical cooling. Nitrogen (N_2_, 100 mL/min) was used as a purge gas. Calibration of the instrument was done with indium and zinc. The reaction enthalpy for different Al/P molar ratios could be obtained by means of Differential Scanning Calorimetry in a temperature scan, and by using stainless steel, reusable, high-pressure crucibles.

The heat flow of the pastes over time was recorded using TAM Air isothermal micro-calorimeter (TA Instruments, Dallas, TX, USA). A temperature of 60 °C was used in order to accelerate the procedure, since MKPC is a rather slow setting system (approximately two days at room temperature.) The fresh paste was mixed for about 3 min in the ampoule using a Heidolph RZR2041 overhead mechanical stirrer (Sigma-Aldrich, Overijse, Belgium) at each maximum speed, and immediately afterwards, it was placed within the calorimeter channels. In total, approximately the first 40 min of the reaction were not monitored due to time loss while preparing the samples and in the period of reaching the equilibrium of the instrument. The heat flow was recorded for the first 72 h of the reaction, and the heat flow values were normalized by the total weight of the paste, the weight of metakaolinite, as well as weight of phosphates and water in the system. The water percentage was kept constant for all the different series of experiments. Based on the heat evolution curves and the reaction enthalpy collected for each different Al/P molar ratio, an estimation of the one which could give optimal properties could be made.

Infrared spectroscopy was performed on a Nicolet 6700 FT-IR Spectrometer (Thermo Fisher Scientific, MA, USA). Parts of the fresh mixture, as well as powder from MKPC hardened cement pastes were placed directly on the ZnSe plate of the smart Attenuated Total Reflectance (ATR) Sampling Accessory. Fourier-transform infrared spectroscopy (FTIR) spectra were taken in a range of 4000–600 cm^−1^. For each spectrum, 32 scans with a resolution of 4 cm^−1^ were averaged. Peak areas were modeled to determine the kinetics of the polymerization reaction.

Thermogravimetric analysis (TGA) was performed on hardened cement paste. A TGA Q5000 model (TA Instruments, Dallas, TX, USA), operating in air, was used for analysis in this work. Measurements were carried out on approximately 25 mg of sample mass in alumina crucibles. Initially, the temperature was maintained at 60 °C for 5 min. The temperature was then raised at 10 °C/min up to 1000 °C with a dwell step of 5 min.

The mechanical properties of MKPCs were investigated by using an Instron 5900R device (Norwood, MA, USA). The loading was set at a rate of 0.5 mm/min for all measurements. Tests were performed on cylindrical specimens having a diameter of 32 mm and 60 mm height. The specimen edges were cut and polished by using a diamond cutting machine to ensure parallel surfaces and alignment. Four samples of each formulation were tested, and the average compressive strengths were reported. Sample fragments collected from compression tests were used to do the characterization through TG-DSC, FTIR, and SEM.

The phase content of the metakaolinite powder, as well as of the developed inorganic cement, was investigated by X-ray powder diffraction analysis (XRD). Diffractograms were recorded on a D2 diffractometer (Bruker Nederland BV) using CuΚα radiation, an acceleration voltage of 45 kV, a current of 30 mA, a step size of 0.020° and °2θ range from 10° to 70°. Phase identification was carried out with the EVA V.3.1 software (Bruker AXS, the Netherlands).

Finally, the microstructure of MKPC pastes, cured at room temperature, was analyzed with a Phenom Pro Desktop scanning electron microscope (SEM) (Thermo Fisher Scientific, MA, USA)

## 3. Results

### 3.1. Stoichiometry of the Metakaolinite Phosphate Cementitious Binder

To produce large volumes of cementitious materials, it is of great importance to be able to control the reaction and keep it at a constant and low rate. The formation of bonds can be either aggressive, i.e., having highly exothermic reactions, or slow, not leading to full conversion. This can be extensively studied through calorimetry by checking the influence of the Al/P molar ratio on the reaction rate.

**Figure 1:**
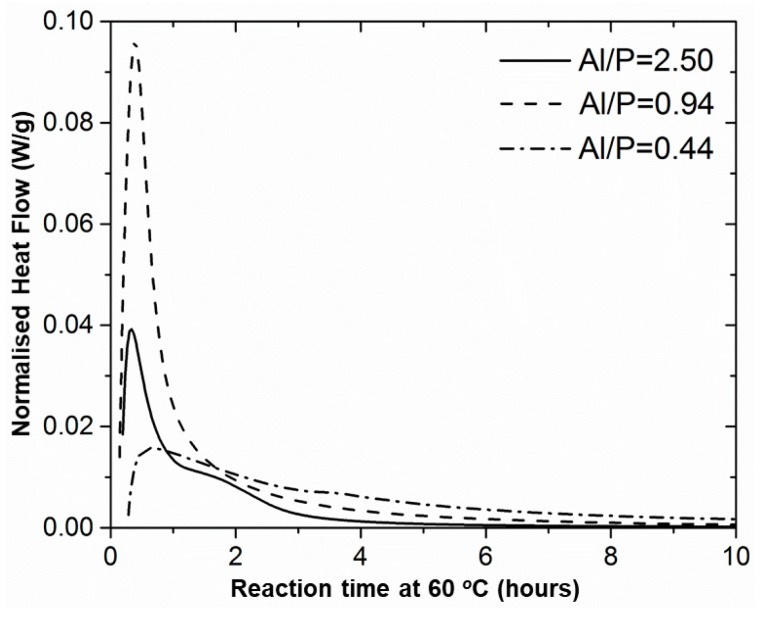
Evolution of reaction enthalpy as a function of Al/P molar ratio. Isothermally, 60 °C, first 10 h of experiment.

To determine the stoichiometry of the reaction and to get an idea of the reaction kinetics, isothermal calorimetry was used (Figure 1, Supplementary Figure S1). The ratio metakaolinite over phosphoric acid solution was varied from 0.44 to 2.50 while keeping the quantity of water in the system constant. The experiments were performed at 60 °C to accelerate the hardening. By integrating the heat flow developed during the experiment (until the end of the reaction), the total heat of reaction was obtained.

**Figure 2:**
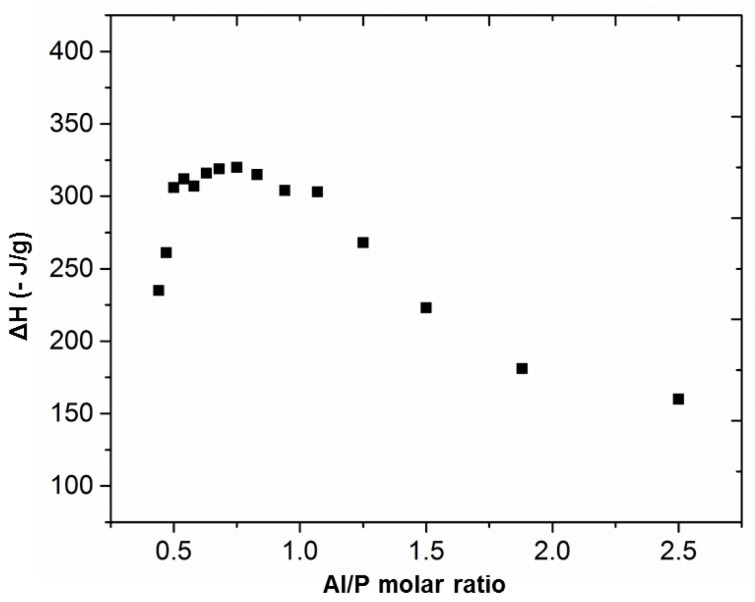
Evolution of the reaction enthalpy as a function of Al/P molar ratio, by means of isothermal calorimetry, 60 °C.

The obtained results revealed that by increasing the amount of phosphoric acid solution, hence decreasing the Al/P molar ratio, the reaction heat also increased until a maximum value around −320 ±1.4 J/g in the range of 0.6 < Al/P < 1.1 (Figure 2). Each (AlO_4_) tetrahedron introduces a negative charge which can be balanced by the positively charged phosphate group (PO_4_). Thus, at an Al/P molar ratio of 1, they are balanced, which can explain the maximum reaction heat in that region. This composition is consistent with that of berlinite [25]; however, it is not sure that berlinite can be formed at room temperature. In the range of 0.6 < Al/P < 1.1 a plateau was reached. By exceeding this molar ratio, the reaction heat decreased again. The fact that no clear maximum but a plateau was obtained could be due to an incomplete reaction. However, the stoichiometric composition was within an Al/P molar ratio within the 0.6 < Al/P < 1.1 range. Thus, an estimation of the composition that could give the best mechanical properties was achieved.

**Figure 3:**
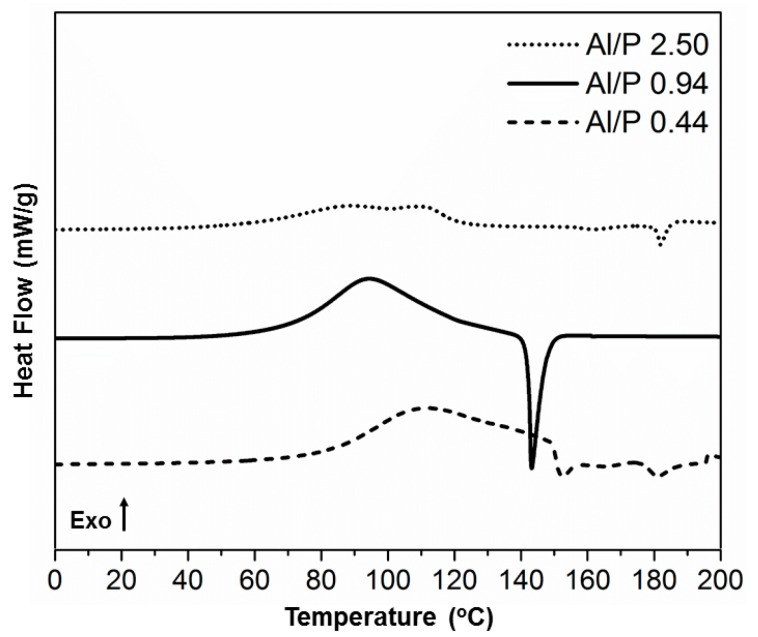
DSC thermograms of the reaction of metakaolinite with phosphoric acid with variation of Al/P molar ratios.

The shape of the exothermic reaction during heating of the reaction mixtures (Figure 3) largely depends on the Al/P molar ratio. For a high value (low phosphoric acid content), a double exotherm was observed. For a value of around one and below, only one exotherm was evident, but it shifted to higher temperatures with decreasing Al/P molar ratios. At the end of the reaction, an endotherm was visible, which was investigated with TGA (see further). In a second heating, no transitions could be detected in this temperature range.

The endotherm increased in size with the reaction exotherm as the Al/P ratio increased, at least in the range of the plateau. A constant ratio between exothermic and endothermic reactions was found in this range. The fact that this ratio was constant meant that it was the reaction product (the exotherm) that underwent a transition (the endotherm). In the DSC thermogram with Al/P = 0.94, close to what is supposed to be the stoichiometric ratio, the endotherm, therefore, was large and sharp, which is in agreement with the large exotherm.

In many of the thermograms, there was an overlap between the first exothermic and endothermic phenomena, which was the reason why these DSC thermograms could not be used to accurately calculate the reaction enthalpies as was done using isothermal calorimetry (Figure 2).

In the infrared spectra of most of the aluminosilicate materials, two vibrational frequency regions are of great importance [36]. The first is between 4000 and 3000 cm^−1^ and is related to stretching vibrations of adsorbed water or OH groups, known as the functional group region; the second one is between 1400 and 600 cm^−1^, linked to Al–OH and Si–O vibrations.

**Figure 4:**
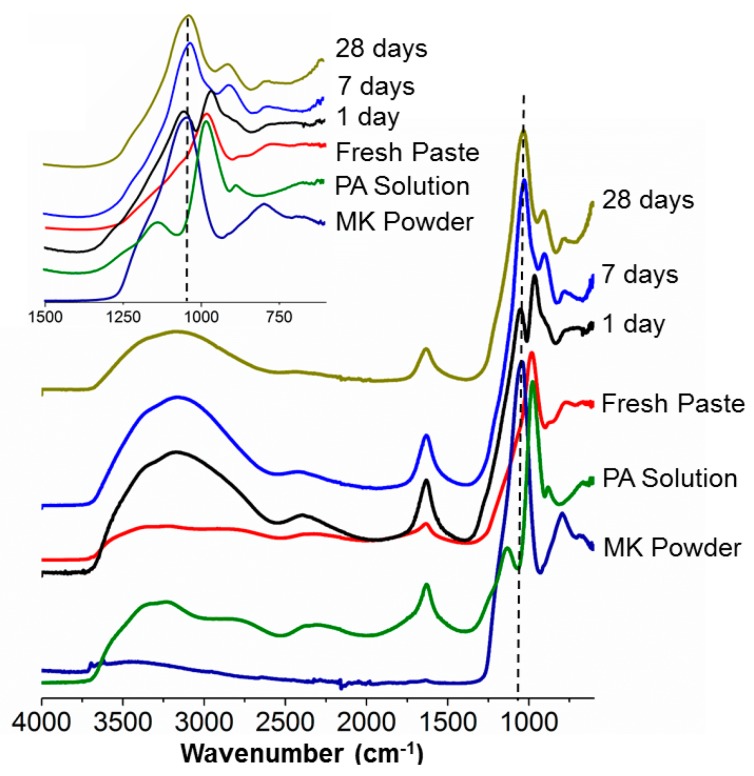
Infrared absorption spectra of commercial MK used as the raw precursor; diluted PA solution used for the synthesis of MKPC; and of MKPC (Al/P = 0.94), cured at room temperature, recorded at various curing ages (as a fresh paste, after 1, 7, and 28 days).

The spectrum of MK (Figure 4) was characterized by the presence of remaining hydroxyl groups, an indication of the incomplete calcination of the kaolinite. The peak at 1042 cm^−1^ was related to asymmetric SiO–Al and Si–O–Si stretching, and the absorptions at 795 cm^−1^ and 691 cm^−1^ revealed the presence of quartz. The spectrum of the H_3_PO_4_ solution that was used to synthesize the optimum composition of the MKPC was characterized by broad peaks at 3231, 2800, and 2298 cm^−1^, assigned to P–OH (3231 cm^−1^) and O=P–OH stretching, respectively. The absorption at 1633 cm^-1^ was a bending mode of water, most probably overlapping with the O=P–OH stretching. The most intense absorption at 976 cm^−1^ was related to P–OH bending. From MK powder to the fresh paste, the 1042 cm^−1^ band developed towards a shoulder next to the P–OH stretching band (976 cm^−1^) of phosphoric acid. After one-day curing, a new absorption arose from this broad one. Upon further reaction, the new absorption increased in intensity at the expense of the one at 976 cm^−1^, and both signals shifted towards lower wavenumbers. In the case of MKPC after 7 and 28 days, there was a third peak rising at about 800 cm^−1^, probably linked to berlinite (AlPO4) or another phosphate. The typical broad P–OH and O=P–OH stretching bands were still visible in the fresh paste and after one day. They disappear when the phosphate groups are integrated in the three-dimensional structure.

**Figure 5:**
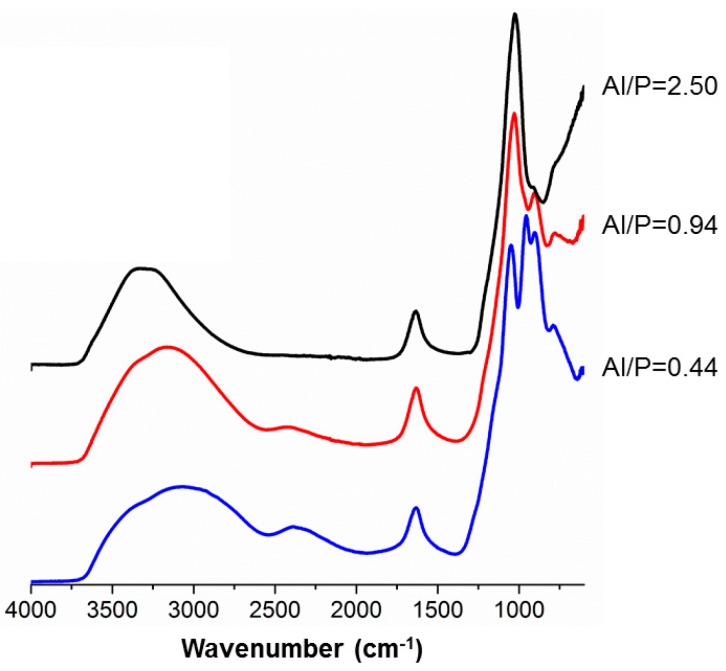
Infrared absorption spectra of MKPC, cured at room temperature, with different Al/P molar ratios, seven days after synthesis.

Next, the influence of different Al/P molar ratios on the MKPC molecular structure was studied. (Figure 5) The spectrum related to Al/P = 0.44 resembled that of pure phosphoric acid, but the absorptions of the pure H_3_PO_4_ (85%) at 1200 and 1145 cm^−1^ moved towards lower wavenumbers and became shoulders on the main absorption of MK at 1042 cm^−1^. Moreover, the absorption at 903 cm^−1^ shifted towards lower wavenumbers (790 cm^−1^); this stemmed from the excess of phosphoric acid, and underpinned the findings from calorimetry. The Al/P = 0.94 spectrum was distinguished by the Al/P = 2.50 and by the appearance of a new peak at 918 cm^−1^, which probably indicated the formation of a new phase. The broad band of the hydroxyl group shifted to higher frequencies and became sharper when less H_3_PO_4_ was used. The band at around 2400 cm^−1^ even completely disappeared at high AL/P ratios. This indicates that the number of POH groups decreased, even relative to the amount of P.

**Figure 6:**
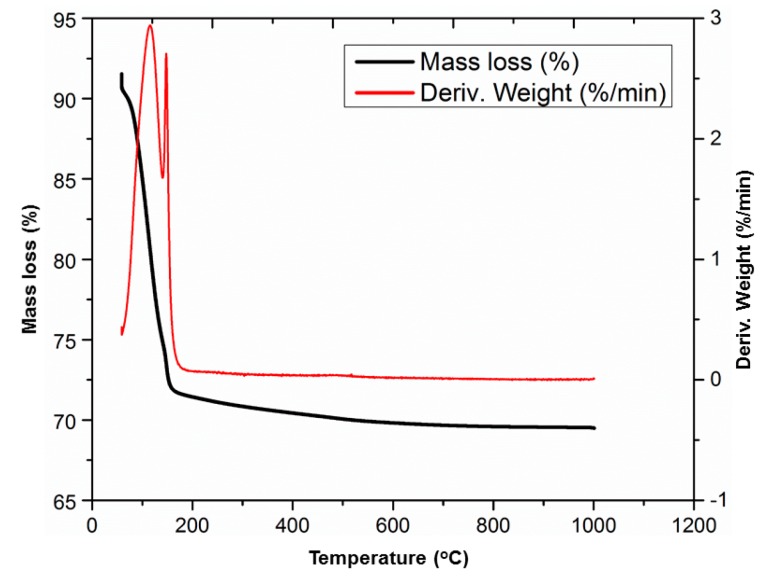
TGA thermogram on the post-cured (1 d at 60 °C) paste with Al/P = 0.94.

TGA of the post-cured paste (1 d at 60 °C) revealed the release of free and bound water (Figure 6). Different stages could be distinguished in the derivative of the mass loss curves. During the reaction, a certain percentage of water became chemically bonded and the rest remained as free water. The free water evaporated from the start of the experiment (14.5%). The distinct mass loss of 13.6% at 127 °C stemmed from a hydrated structure or OH groups. That hydrate water was not clearly visible in FTIR spectra as a sharp peak, meaning that it belongs to an amorphous phase. This chemically-bound water is, however, the origin of the endotherm observed in non-isothermal DSC measurements. The difference in temperature (always higher in DSC) is due to the fact that closed sample pans are used in DSC; thus, the partial pressure of water is higher, shifting the decomposition to higher temperatures. Above 140 °C, the mass loss was much slower, and above 600 °C, the mass remained stable.

**Figure 7:**
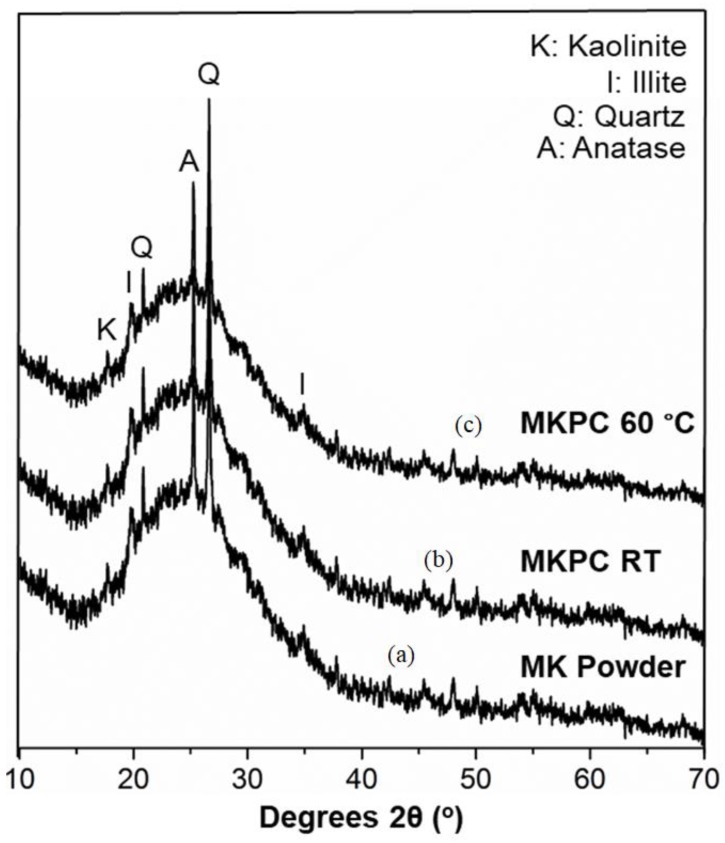
X-ray diffraction patterns of (**a**) metakaolinite, the raw precursor used, (**b**) of the metakaolinite-phosphate cement cured at room temperature, and (**c**) of the metakaolinite-phosphate cement post-cured at 60 °C.

The XRD diffractograms of MKPC revealed the presence of a diffuse halo between 20° and 30°; thus, the formed metakaolinite phosphate cement was mainly amorphous. (Figure 7) Metakaolinite is amorphous, but crystalline phases of quartz, anatase, and illite are present in this sample. According to previous studies, phases like illite, quartz, gypsum and hematite are minerals associated with kaolinite, and in principle, they do not participate in the chemical reaction [23,37]. After heat treatment, the reflections related to kaolinite disappeared due to the formation of metakaolinite [26,30]. The unfired MKPC (cured at RT) showed only small reflections of quartz. After post curing at 60 °C, the material remained highly amorphous, with no differences in the diffractograms.

### 3.2. Microstructure

**Figure 8:**
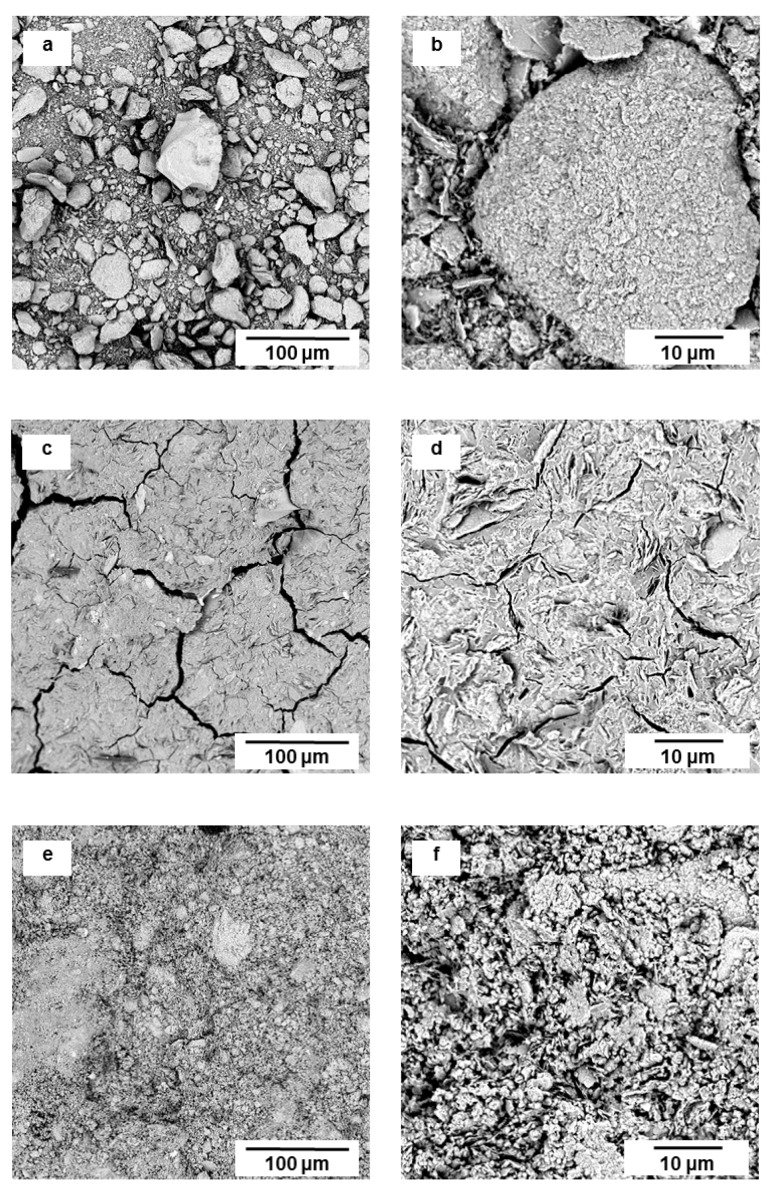
SEM micrographs of MKPC pastes (without sand) cross sections at different Al/P molar ratios: (**a**,**b**) Al/P = 0.44; (**c**,**d**) Al/P = 0.94; (**e**,**f**) Al/P = 2.50. (Cured at room temperature, 28 days after synthesis)

Microstructure and porosity are considered as critical factors for the mechanical properties of cementitious materials. The microstructures shown in Figure 8 correspond to three different Al/P molar ratios (Al/P = 0.44; Al/P = 0.94; and Al/P = 2.50 respectively) cured at room temperature. The presence of micro-cracks and the rough surface, which exist in the paste’s micrograph with Al/P = 0.94 and Al/P = 2.50, may be related to the fact that unpolished fragments were used for analysis. Thus, the structure contained cracks created through the fragmentation of the specimens. The sample with Al/P = 0.44 did not harden because of its high phosphate concentration. The specimen was washed with distilled water and the powder was filtered and dried before SEM testing. Compared to MK, next to small particles, also larger beads are seen, consisting of reacted material. MKPC with Al/P = 0.94 shows a plate-like structure glued together by the matrix Figure 8c,d. In the sample with Al/P = 2.50, the plate-like structure is also revealed, but much less binding matrix is present. This clearly shows that the closer the Al/P molar ratio is to one, the denser the structure of the obtained MKPC.

### 3.3. Mechanical Properties

**Figure 9:**
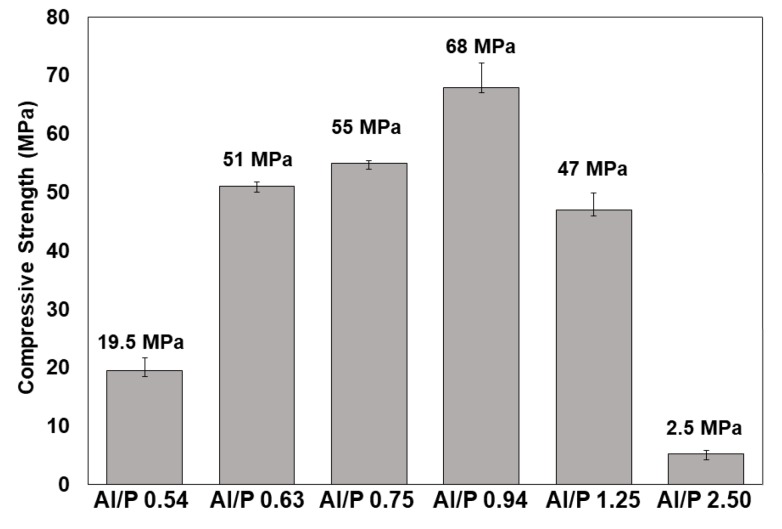
Evolution of the mechanical strength of studied MKPC mortars as a function of Al/P ratio. Mortars were cured for 1 day at room temperature, and post-cured at 60 °C for another day. They were mechanically tested seven days after synthesis.

The average strength of mortars increased with increasing Al/P molar ratio up to 68 MPa, seven days after synthesis (including 24 h at 60 °C), for an Al/P molar ratio of 0.94 (Figure 9). The compressive strength of the Al/P = 0.94 mortars at 14 and 28 days was 67 ± 6 MPa and 69 ± 2.5 MPa respectively, indicating their ageing stability. This could be expected in view of the accelerated hardening at 60 °C. At higher molar ratios, the compressive strength gradually decreased. These results were in line with the calorimetry, predicting an optimum between 0.6 and 1.1. The decrease of mechanical strength above and beneath the Al/P ratio of 1 was, however, not only due to porosity. Specimens with such Al/P ratios were not stable and suffered a decrease in strength over time. For example, specimens with Al/P = 0.75 molar ratio were characterized by 55 MPa average mechanical strength at seven days after casting, which, however, dropped to 47 and 36 MPa for fourteen and twenty-eight days post-curing time, respectively.

The water content, and thus, porosity, can still be lowered, but in view of the application for textile reinforced cements with over 20 vol% fibers, a good workability is needed. However, a too high-water content causes excessive cracks and shrinkage. The optimal can be achieved if the water content is low but the viscosity of the slurry is still in between acceptable limits to have good workability. The water stability of the synthesized specimens with the optimum molar ratio of Al/P = 0.94 was tested by immersing the specimens in water for long periods (1 year); the specimens were proven to be stable in water.

## 4. Discussion

The study of the reaction mechanism and the reaction stages involved in the MK/H_3_PO_4_ system is complex, especially for the Al/P molar ratios close to the optimum. In literature, several reaction mechanisms have been described, with most researchers agreeing with Davidovits and his assumption that chemically bonded phosphate cements behave as geopolymers (alkali-activated cements) and follow the same reaction mechanism [38]. Guo et al. suggested a simplified explanation where the reaction stages mentioned for such systems are the dissolution, polymerization, and polycondensation [39]. The existence of these steps could, however, not be proven. Based on the presented results (Figure 1), only for the extreme Al/P = 2.50 curve, two overlapping exothermic peaks can clearly be distinguished, where the second peak could be attributed to the polymerization reaction. For the other molar ratios, only one main peak was present, indicating that the first stage of exothermic dissolution started immediately when the sample was mixed, and the maximum was always observed during the first hour of reaction. The large exothermic peak in the beginning of the measurements corresponded to a rapid dissolution process of the reactive aluminosilicate particles into the acidic environment, but probably the polymerization reaction overlapped with the dissolution. As such, they cannot be seen as separate signals.

Both calorimetry and mechanical testing support the hypothesis that the stoichiometric ratio is obtained at Al/P equals 1. This coincides with the charge balancing of the (AlO_4_)^−^ groups by (PO_4_)^+^ groups from the activating solution. It is also comparable to the charge balancing observed in alkali-activated aluminosilicates where the negative charge of the (AlO_4_)^−^ groups is compensated by the positive charge of the cation from the activating solution [40].

Several researchers mention the presence of an AlPO_4_ phase (cristobalite or tridymite type) in aluminum and/or aluminosilicate based phosphate cements [30,39]. They state that AlPO_4_ is formed during the step of dealumination (at room temperature) by the reaction between the leached Al^3+^ species and (PO_4_)^3−^ units of phosphoric acid. Berlinite was, however, only detectable after thermal treatment [24,33,41,42,43]. AlPO_4_ is indeed formed by the reaction between phosphoric acid and alumina. However, berlinite is isomorphous with various forms of silica; thus, mixed aluminium and silicon phosphates might also be formed [44]. Also, both qualitative and quantitative XRD analysis did not identify that phase. Although the berlinite was pure and crystalline, still other absorptions were expected in IR, but these could not be retrieved. The formation of berlinite during the reaction, therefore, remains to be proven. It was not possible with the analyses performed to find out if Al is present only in an AlPO_4_ phase, and thus, that Si from MK formed a silica phase, or if a mixed phospho-alumino-silicate had been formed [45]. The shift of the most intense absorption in IR from 1042 cm^−1^ (MK) to 1033 cm^−1^ after 28 days curing may also be due to a combination of silicon, aluminium and phosphorous oxides [36].

The decrease in strength upon aging for specimens with the Al/P ratio below 1 shows that such ratios have an excess of phosphoric acid that cannot react with MK. The excess then breaks down the formed binder. An excess will already occur at a sub-stoichiometric ratio. This is clear from the calorimetry results (Figure 2), where a plateau value is observed near the optimum rather than a distinct maximum. Thus, a fraction of the reagents does not react (the extent of reaction remains below 100% with consequently only partially reacted MK). Moreover, by increasing the concentration of the hardening solution and reaching the stoichiometric value, the percentage of unreacted phosphorous acid will also increase. This can have a severe impact on the mechanical properties of the final material, or even cause degradation of the structure after setting. The degradation was reported in literature by Rüscher et al. [46], referring to the weakening of the structure of alkali activated cements when the stoichiometric ratio is not the optimum [40]; thus, an excess of activator leads to deterioration of the structure.

The development of this matrix was done in view of the production of textile-reinforced cements. The acidic matrix which becomes neutral makes possible the use of cheap E-glass fibers as reinforcements. These composites will be studied in future papers.

## 5. Conclusions

Metakaolinite phosphate cements were synthesized from metakaolinite, phosphoric acid, and distilled water. We have shown that metakaolinite phosphate cements with Al/P ratios close to 1/1 are optimal. However, although MKPCs with Al/P > 1 are viable, they lose strength and microstructure stability. For Al/P ratios < 1, the resulted material contains excessive amounts of water and phosphates which reduce the capabilities of the material. Based on the calorimetric results, a plateau is reached, identifying the range where the optimum composition is found, as well as an incomplete reaction. Reaction at room temperature takes seven days at least, and unreacted PA remains in the structure. According to calorimetry and thermogravimetry, a hydrated structure is formed at about 127 °C. The compressive strength of the newly-developed cement is >60 MPa, reaching to a maximum of 68 MPa on average. In addition to the above, we also demonstrated the importance of the raw material’s properties and the concentration of the hardening liquid, which are believed to be the most critical factors for the developed system. Hardened specimens have been shown to be water stable by leaving them immersed in water for extended periods (>1 year) to ensure their dimensional stability.

## Figures and Tables

**Table 1:** Chemical composition of metakaolinite and phosphoric acid in wt%.

Compositions (Weight %)								
	SiO_2_	Al_2_O_3_	Fe_2_O_3_	TiO_2_	K_2_O + Na_2_O	CaO + MgO	P_2_O_5_	LOI
Metakaolinite	55	39	1.8	1.5	1	0.6	-	1
H_3_PO_4_	-	-	-	-	-	-	85	-

## Supplementary Materials

The following are available online at http://www.mdpi.com/1996-1944/12/3/442/s1, Figure S1: Evolution of reaction enthalpy as a function of additional Al/P molar ratios. Isothermally, 60 °C, first ten hours of the experiment.
