# Supplementary figures and images for: Metakaolinite Phosphate Cementitious Matrix: Inorganic Polymer Obtained by Acidic Activation
Source: Materials (Basel). 2019 Jan 31;12(3):442. doi: 10.3390/ma12030442 (PMC6384563; doi:10.3390/ma12030442)

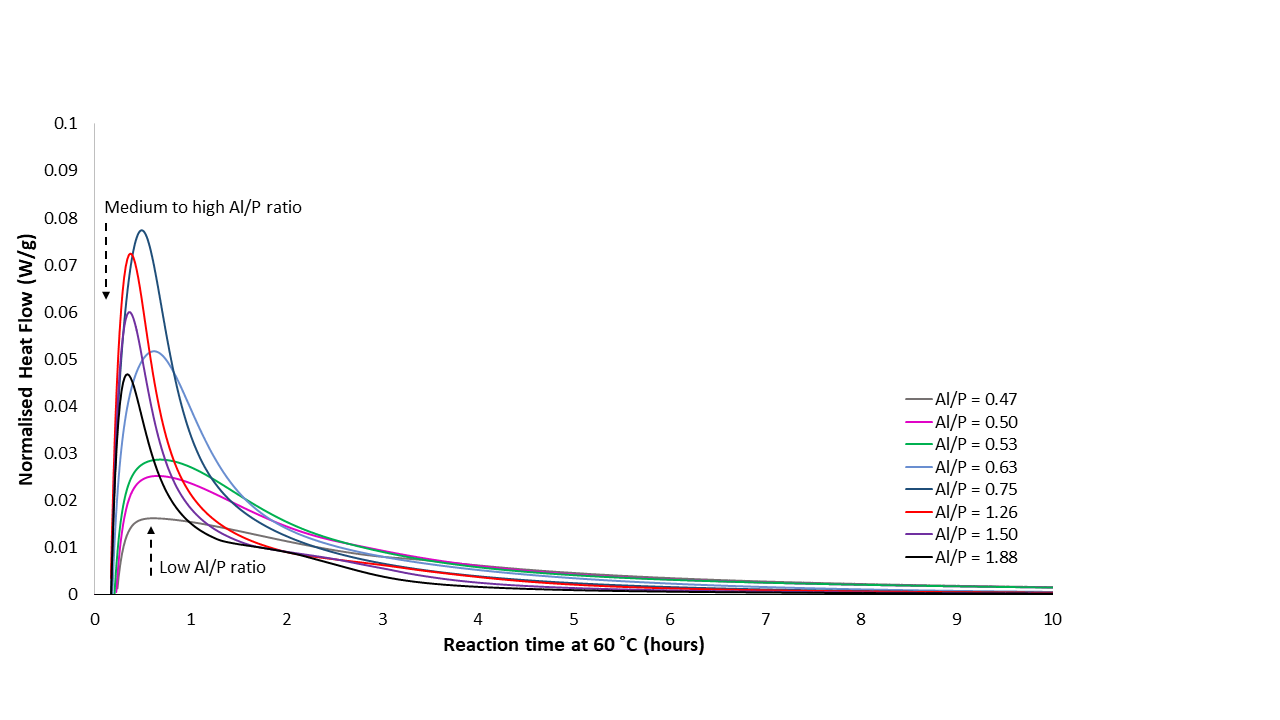

Supplement: Supplementary file 1 [file materials-12-00442-s001.zip › materials-423465-supplementary.tif]
